# Over-the-Counter Hearing Aids Challenge the Core Values of Traditional Audiology

**DOI:** 10.1044/2023_JSLHR-23-00306

**Published:** 2024-02-08

**Authors:** Katherine N. Menon, Michelle Hoon-Starr, Katie Shilton, Eric C. Hoover

**Affiliations:** a Department of Hearing and Speech Sciences, University of Maryland, College Park; b College of Information Studies, University of Maryland, College Park

## Abstract

**Purpose:**

Regulatory changes in the United States introduced over-the-counter (OTC) hearing aids with the goal of increasing the accessibility and affordability of hearing health care. It is critical to understand the values inherent to hearing health care systems to evaluate their effectiveness in serving people with hearing difficulty. In this study, we evaluated the relative importance of values across service delivery models and the extent to which the introduction of OTC hearing aids represents a values shift relative to traditional audiology.

**Method:**

We performed a qualitative content analysis of two document categories: critique documents that motivated the creation of OTC hearing aids and regulatory documents that defined OTC hearing aids. Team members coded portions of text for the values they expressed. In total, 29,235 words were coded across 72 pages in four documents. Rank-order analyses were performed to determine the prioritization of values within each category of documents and subsequently compare values between OTC and traditional audiology documents analyzed in a previous study.

**Results:**

Critique and regulatory documents both prioritized values related to reducing barriers to hearing aid access and use, but the lack of a significant correlation in the rank order of values in these documents was evidence of inconsistency between the motivation and implementation of OTC hearing aids. Differences in the rank order of values in the OTC documents compared to traditional audiology were consistent with a values shift.

**Conclusions:**

The introduction of OTC as a solution to low hearing aid use represents a values shift, challenging the values of traditional audiology. This research demonstrates a need to establish the values of hearing health care service delivery through a consensus of stakeholders, including individuals from diverse backgrounds underserved by the traditional model.

Hearing loss is the third most common chronic health condition among Americans, affecting approximately two-thirds of adults over 70 years (Bainbridge & Wallhagen, 2014). Despite the high prevalence of hearing loss and its associated consequences, less than 30% of adults who could benefit from amplification devices seek them (Dammeyer et al., 2017). Hearing aid adoption is a complex issue that may be affected by a variety of individual factors, such as self-perceived degree of hearing impairment, technology commitment, socioeconomic status, psychomotor function, and self-reported health status (Jorgensen & Novak, 2020; Nixon et al., 2021; Tahden et al., 2018). Systemic factors also mediate the use of hearing aids among those who could benefit, and the National Institute on Deafness and Other Communication Disorders made it a goal to understand and eliminate these systemic barriers (Donahue et al., 2010). Two high-profile expert analyses of hearing health care in the United States identified broader systemic forces that contribute to untreated hearing loss, published by the President's Council of Advisors on Science and Technology (PCAST, 2015) and the National Academies of Sciences, Engineering, and Medicine (NASEM, 2016). Both reports provided an evidence-based critique of current hearing health care practices in the United States and identified several issues that might contribute to the apparent preference for nonuse of hearing aids. The reports concluded that lack of access to a hearing health care professional and the high cost of devices are the most significant barriers to adopting hearing aids. To overcome issues related to access and affordability, the expert analyses recommended that the Food and Drug Administration (FDA) introduces a new class of hearing aids that can legally be sold direct to consumer. Legislation was subsequently passed in 2017 that introduced a new class of amplification devices to be sold over the counter (OTC; Over-the-Counter Hearing Aid Act of 2017, passed as part of the FDA Reauthorization Act HR 2430, §934 2017, of U.S. Congress, 2017). In 2022, the FDA issued a finalized rule establishing the legalization of OTC hearing aids (U.S. FDA, 2022). Beginning October 17, 2022, adults over the age of 18 years with perceived mild-to-moderate hearing loss were enabled to independently purchase hearing aids without consulting a hearing health care professional.

OTC hearing aids were explicitly justified as an effort to alleviate issues identified in the PCAST and NASEM reports (Warren & Grassley, 2017), but they are not a panacea. Multifaceted, individual, and systemic factors contribute to the low rates of hearing aid use by people who could benefit from them, and OTC hearing aids address a subset of the systemic barriers identified in the reports. OTC hearing aids are an opportunity for the market to produce effective solutions. The development of effective solutions leveraging the OTC model will require further examination of the objectives that motivated the regulatory changes. We can use values as a framework to evaluate and critique novel hearing health care solutions, whether those solutions reflect the OTC model or the traditional model of hearing health care, where traditional audiology refers to hearing health care provided by a hearing health care professional in the United States. Furthermore, values can be used to understand how the introduction of the OTC model represents a challenge to the values of traditional audiology, which, as the dominant model of hearing health care, was the source of many systemic barriers identified by PCAST and NASEM. Although access and affordability are valued in traditional audiology, accuracy, safety, efficacy, and other values drive the provision of hearing health care in that model (Menon et al., 2023). By gaining a deeper understanding of the values that underlie different hearing health care models, we can potentially reduce unintended adverse consequences that major regulatory changes may have on patients and consumers.

This study leverages value-sensitive design (VSD), an approach for the design of technologies that embody specific values (Friedman et al., 2002, 2013). Values are principles or qualities appreciated as important by an individual or system. VSD assumes that by eliciting values of different stakeholders and identifying values in products and services, we can design solutions that better reflect stakeholder values. In recent years, VSD has been applied in health care research to inform both the development of ambulatory therapy technologies for patients in their homes and information and communication technologies for patients with chronic diseases (Dadgar & Joshi, 2018; Mueller & Heger, 2018). Our work is the first to apply this methodology to hearing health care (Menon et al., 2023). The goal of this work is to leverage values to encourage the millions of people who could benefit from amplification to obtain the services they need or to reduce the delay in getting those services (Simpson et al., 2019). This will be accomplished by matching the values of hearing health care products and services to the values of underserved patient populations. First, it is necessary to understand the values inherent to the systems of hearing health care that are designed to serve people with hearing difficulty.

Our recent work developed a comprehensive list of values in traditional audiology through an iterative textual analysis of documents representing the ethics, procedures, and outcome measures used in audiology clinical practice (Menon et al., 2023). In this study, we analyzed documents representing traditional, clinical audiology in order to identify the values instantiated by the current dominant model of hearing health care in the United States. Three categories of documents representative of traditional audiology were selected: questionnaires, clinical practice guidelines, and codes of ethics. These documents were selected, because they represent dimensions of *intended* and *enacted* values in traditional audiology (Shilton et al., 2013). Questionnaires represent enacted audiology values, because audiologists use them to assess outcomes and determine treatment success. Clinical practice guidelines are a record of what audiologists do, following best practice recommendations where available, and, therefore, document both dimensions of intended and enacted values in clinical practice. Codes of ethics represent intended values that define moral behavior for audiologists, as determined by professional organizations. Although the documents chosen for analysis did not encompass all aspects of audiology clinical practice, it is unlikely that including additional documents would have expanded the list of values because of the methodology used (Curtis et al., 2001). The final list of values in traditional audiology was divided into three categories: instrumental, patient use, and moral values. Instrumental values included accuracy, cost, design, efficiency, evidence-based, objective benefit, and safety. Patient use values included comfort, ease of use, health, satisfaction, self-efficacy, and subjective benefit. Moral values included access to care, autonomy, privacy, equity, and professional duties. The values identified in traditional audiology documents were then rank ordered according to the number of times each value appeared across documents. The current study applied VSD to hearing health care by identifying values in documents representing the introduction of the OTC model and contrasting them with values in traditional audiology.

Our central hypothesis was that the introduction of OTC hearing aids reflects the prioritization of access and affordability over all other values and the deprioritization of core values of traditional audiology that could compete with access and affordability. This was evaluated with two research questions. The first research question was if the implementation of the OTC model shares the same values as the critique documents that motivated its creation. This was evaluated by comparing the rank order of values in documents representing the critical motivation to create OTC compared to the regulatory documents that defined OTC hearing aids. The similarity of values rankings between these documents was used to determine the extent to which the introduction of OTC hearing aids represented a coherent values framework that could be contrasted with the values of traditional audiology. The second research question was if the introduction of OTC hearing aids represents a challenge to the values of traditional audiology. This was evaluated by comparing the rank order of values between OTC documents and traditional audiology documents. A positive correlation between the values in OTC and traditional audiology would reject the central hypothesis that the introduction of OTC hearing aids represents a targeted reprioritization of values in hearing health care.

## Method

A summative content analysis (Hsieh & Shannon, 2005; Morse & Field, 1995) was performed to identify the values expressed by the text in two critique documents representing the motivation to create an OTC model (PCAST and NASEM reports) and two regulatory OTC documents representing the implementation of OTC hearing aids in the United States (Final FDA regulations on Establishing OTC Hearing Aids; OTC Hearing Aid Act of 2017). The rank order of values found in documents representing OTC hearing aids were compared to the rank ordered values found in traditional audiology documents reported in Menon et al. (2023). The complete list of the 33 documents representing traditional audiology can be found in the Appendix of Menon et al. The PCAST and NASEM reports represent intended values in hearing health care, because they describe the motivation to create a new hearing health care model. The OTC Law and FDA regulations are our best representations of enacted values in hearing health care, since they describe principles of the model that have been translated into concrete laws, regulations, and policies. It is critically important to analyze the enacted values in OTC hearing aids to ensure that intended values driving the creation of the system manifest in the actual implementation of the model.

Two team members independently read materials and methodically assigned meaning to text data by tagging relevant passages with the appropriate value(s), a process referred to as coding (Locke et al., 2020; Saldaňa, 2009). The codebook of values in hearing health care developed in Menon et al. (2023) was used to guide our analysis. The codebook contains a comprehensive list of values that includes a definition, a short description, and examples of how that value appears in the text. Four documents (listed in Table 1) were analyzed using NVivo 12 Pro (QSR International Pty Ltd., 2018), a software program that facilitates the textual analysis of unstructured documents. This tool was used to identify sections of content in which the meaning of the text corresponds to one or more values as defined in the codebook.

**Table 1.** Source material for qualitative content analysis.

Source material	Category	Represents
Over-the-Counter Hearing Aid Act of 2017	OTC regulation	OTC implementation
Final FDA regulations on Establishing Over-the-Counter Hearing Aids (2022)	OTC regulation	OTC implementation
NASEM Report (2016)	Critique document	Motivation to create OTC
PCAST Report (2015)	Critique document	Motivation to create OTC

*Note.* OTC = over the counter; FDA = Food and Drug Administration; NASEM = National Academies of Sciences, Engineering, and Medicine; PCAST = President's Council of Advisors on Science and Technology.

Following independent coding, the data were merged via consensus. Passages with nontrivial disagreement between the two coders were discussed until a consensus was reached. The use of multiple coders allows for broader possibilities for interpreting and understanding data (Fonteyn et al., 2008) and ensuring intercoder agreement improves consistency and accuracy of results (Fernald & Duclos, 2005). After coding was complete, quantitative data were extracted from the software to compare the relative importance of values within and between documents. There are multiple ways to quantify the results of the coding process. For this study, we calculated the *frequency* of each value by counting the total number of words coded to that value. A total word count was used instead of a proportional index, because the documents differed greatly in length and scope, making a proportional index weigh too highly the values relevant to the scope of the shorter documents. We operationally defined the priority of a value as the frequency of coding references to that value in the text. By defining coding frequency, and thus importance, as the number of words coded, we are assuming that the authors of the documents dedicated more text to issues that they valued more.

## Statistical Analysis

Kendall's rank correlation was used to test the similarity of the rank order of values. The correlation between rankings was determined at the alpha criterion of α = .05. Kendall's rank distance was used to quantify the magnitude of the difference between documents in the importance of values. Rank distance counts pairwise differences between ranked lists. If one value is ranked above another value in a document, that pair of values would add to the ranked distance if they appeared in the opposite order in a comparison document. A normalized rank distance of zero indicates that the two lists are identical and one indicates that the lists are in reverse order.

## Results

**Figure 1.**
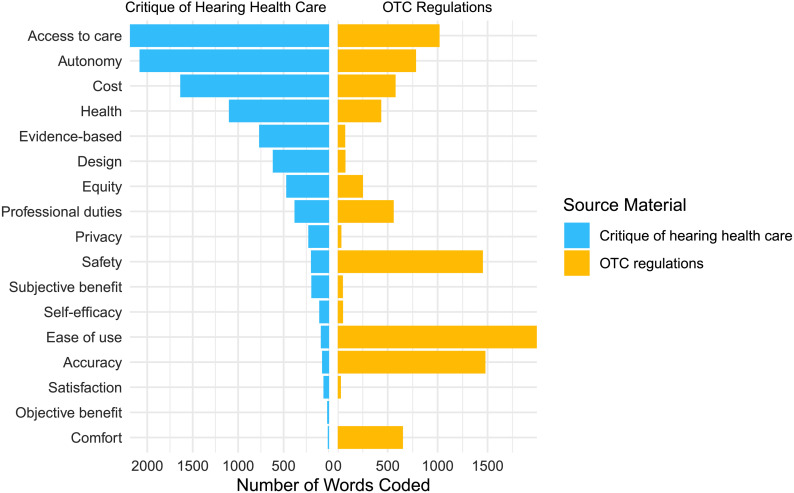
Frequency of each coding reference representing values in critique documents (President's Council of Advisors on Science and Technology, 2015 and National Academies of Sciences, Engineering, and Medicine, 2016) and regulatory OTC documents (OTC Law 2017 and Food and Drug Administration OTC Regulations, 2022), sorted by the frequency of values coded in the critique documents. OTC = over the counter.

The first goal of this study was to evaluate if the documents that represent the implementation of OTC share the same values as the critique documents that motivated its creation. Figure 1 shows the frequency of values in each of the analyzed OTC documents. Table 2 shows the rankings of the number of coding references for each value in critique documents and OTC regulatory documents. Kendall's rank correlation revealed no statistically significant correlation between the ranks of referenced values in regulatory and critique documents (τ = .294, *p* = .096). The normalized Kendall rank distance (*K*_d_) was calculated to evaluate the number of misaligned pairs between these two ranked lists. The result was *K*_d_ = .353, indicating that approximately 35% of code pairs were in the reverse order in the critique documents compared to the OTC regulatory documents.

**Table 2.** Rankings of the number of coding references for each value in critique documents and OTC regulatory documents.

Value	Source material Rank (number of words coded)					
	PCAST	NASEM	Total critique documents	OTC bill	FDA regulations	Total OTC regulations
Access to care	3 (748)	1 (1,442)	**1** (2,190)	2 (200)	4 (819)	**4** (1,019)
Accuracy	15 (16)	12 (60)	**14** (76)	6 (15)	2 (1,463)	**2** (1,478)
Autonomy	1 (911)	2 (1,174)	**2** (2,085)	1 (204)	6 (579)	**5** (783)
Comfort		16 (13)	**17** (13)		5 (652)	**6** (652)
Cost	2 (866)	3 (771)	**3** (1,637)		7 (578)	**7** (578)
Design	5 (583)	14 (35)	**6** (618)		11 (77)	**11** (77)
Ease of use	12 (34)	13 (56)	**13** (90)	5 (25)	1 (1,968)	**1** (1,993)
Equity	11 (39)	6 (431)	**7** (470)		10 (252)	**10** (252)
Evidence-based	7 (139)	4 (630)	**5** (769)		12 (74)	**12** (74)
Health	4 (642)	5 (459)	**4** (1,101)	4 (34)	9 (401)	**9** (435)
Objective benefit	14 (20)		**16** (20)			
Privacy	8 (113)	8 (115)	**9** (228)		15 (36)	**15** (36)
Professional duties	6 (243)	7 (136)	**8** (379)		8 (560)	**8** (560)
Safety	10 (95)	10 (103)	**10** (198)	3 (41)	3 (1,412)	**3** (1,453)
Satisfaction	13 (28)	15 (33)	**15** (61)		16 (31)	**16** (31)
Self-efficacy		9 (107)	**12** (107)		13 (52)	**13** (52)
Subjective benefit	9 (104)	11 (90)	**11** (194)		15 (51)	**14** (51)
Total words coded (*N*)	4,581	5,655	**1,0236**	519	9,005	**9,524**

*Note.* Bolded values indicate total rankings across document groups. PCAST = President's Council of Advisors on Science and Technology; NASEM = National Academies of Sciences, Engineering, and Medicine; OTC = over the counter; FDA = Food and Drug Administration.

**Figure 2.**
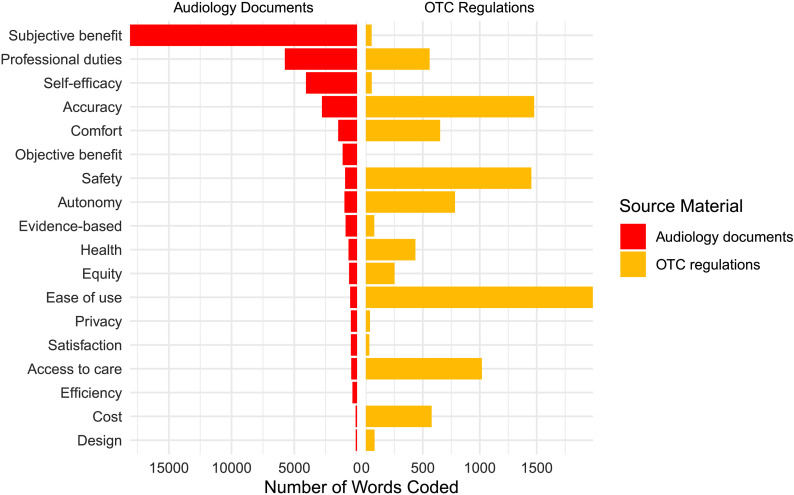
Frequency of each coding reference representing values in traditional audiology documents (clinical practice guidelines, codes of ethics, and questionnaire) and regulatory OTC documents (OTC Law 2017 and Food and Drug Administration OTC Regulations, 2022). OTC = over the counter.

The second goal of this study was to evaluate whether documents representing the OTC model share the same values as documents that represent the traditional audiology model. The results of the first analysis showed that the critique and regulatory documents prioritized values differently, so each set of documents was compared to traditional audiology separately. Figure 2 shows the number of words coded to each value in OTC and traditional audiology documents, and Table 3 shows the ranked order of values found in OTC regulation documents compared to the ranked order of values found in documents that represent traditional audiology (data from Menon et al., 2023). Calculation of Kendall's τ revealed no statistically significant correlation between the ranks of referenced values in regulatory and critique documents (τ = .072, *p* = .709). The normalized *K*_d_ was also calculated to evaluate the number of misaligned pairs between these two ranked lists. We found that *K*_d_ = .464, indicating that approximately 46% of code pairs were in the reverse order in the OTC regulatory documents compared to traditional audiology documents.

**Table 3.** Rankings of the number of coding references for each value in traditional audiology and over-the-counter (OTC) regulatory documents.

Value	Source material Rank (number of words coded)	
	Traditional audiology documents	Total OTC regulations
Access to care	**15** (463)	**4** (1,019)
Accuracy	**4** (2,800)	**2** (1,478)
Autonomy	**7** (1,003)	**5** (783)
Comfort	**5** (1,504)	**6** (652)
Cost	**17** (115)	**7** (578)
Design	**18** (104)	**11** (77)
Ease of use	**12** (549)	**1** (1,993)
Efficiency	**16** (366)	
Equity	**11** (637)	**10** (252)
Evidence-based	**9** (913)	**12** (74)
Health	**10** (677)	**9** (435)
Objective benefit	**6** (1,148)	
Privacy	**13** (495)	**15** (36)
Professional duties	**2** (5,755)	**8** (560)
Safety	**8** (954)	**3** (1,453)
Satisfaction	**14** (494)	**16** (31)
Self-efficacy	**3** (4,070)	**13** (52)
Subjective benefit	**1** (18,082)	**14** (51)
**Total words coded (*N*)**	**40,139**	**9,524**

*Note.* Bolded values indicate total rankings across document groups.

**Figure 3.**
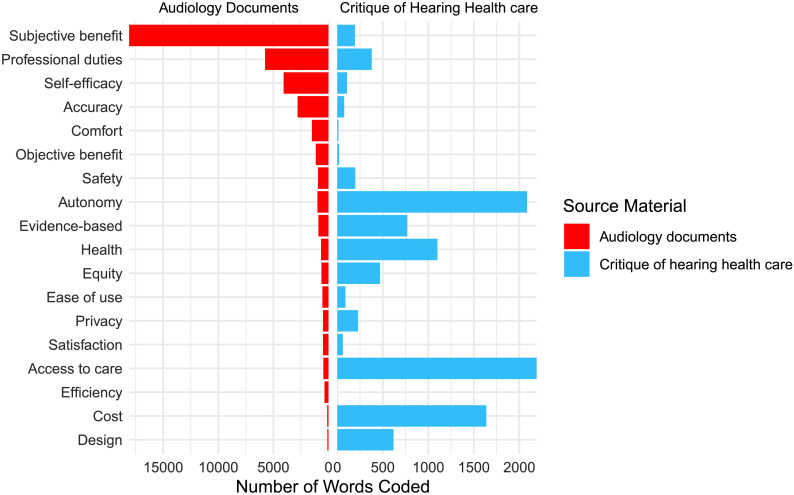
Frequency of each coding reference representing values in traditional audiology documents (clinical practice guidelines, codes of ethics, and questionnaire) and critique documents (President's Council of Advisors on Science and Technology and National Academies of Sciences, Engineering, and Medicine reports).

The PCAST and NASEM reports were evaluated to determine whether the critique of audiology that motivated the development of OTC shared the same values as traditional hearing health care. Figure 3 shows the number of words coded to each value in critique documents and traditional audiology documents, and Table 4 shows the ranked order of values found in critique documents compared to the ranked order of values found in documents that represent the traditional audiology (data from Menon et al., 2023). Calculation of Kendall's τ revealed no statistically significant correlation between the ranks of referenced values in audiology and critique documents (τ = −.137, *p* = .454). The normalized *K*_d_ was also calculated to evaluate the number of misaligned pairs between these two ranked lists. We found that *K*_d_ = .569, indicating that approximately 57% of code pairs were in the reverse order in the OTC regulatory documents compared to traditional audiology.

**Table 4.** Rankings of the number of coding references for each value in traditional audiology documents and critique documents.

Value	Source material Rank (number of words coded)	
	Traditional audiology documents	Total critique documents
Access to care	**15** (463)	**1** (2,190)
Accuracy	**4** (2,800)	**14** (76)
Autonomy	**7** (1,003)	**2** (2,085)
Comfort	**5** (1,504)	**17** (13)
Cost	**17** (115)	**3** (1,637)
Design	**18** (104)	**6** (618)
Ease of use	**12** (549)	**13** (90)
Efficiency	**16** (366)	
Equity	**11** (637)	**7** (470)
Evidence-based	**9** (913)	**5** (769)
Health	**10** (677)	**4** (1,101)
Objective benefit	**6** (1,148)	**16** (20)
Privacy	**13** (495)	**9** (228)
Professional duties	**2** (5,755)	**8** (379)
Safety	**8** (954)	**10** (198)
Satisfaction	**14** (494)	**15** (61)
Self-efficacy	**3** (4,070)	**12** (107)
Subjective benefit	**1** (18,082)	**11** (194)
Total words coded (*N*)	**40,139**	**10,236**

*Note.* Bolded values indicate total rankings across document groups.

## Discussion

### Comparison of Values in Critique Documents and OTC Regulations

We applied the codebook developed in Menon et al. (2023) to systematically assess the values in source material relating to the justification and implementation of OTC hearing aids. Our central hypothesis was that the development of an OTC regulatory category represents a values shift relative to the values of traditional audiology, prioritizing access, and affordability over all other values in hearing health care. The first objective of this study was to compare the frequency of values referenced in critique documents (PCAST and NASEM reports) and regulatory OTC documents (OTC Law and final FDA regulations) to determine whether the implementation of the OTC model shared the same values as the critique documents that motivated its creation. Although there was no significant correlation between the ranks of referenced values, Kendall's rank distance test showed that approximately two-thirds of values were in the same rank order in both documents (35% misaligned), indicating moderate agreement between the two lists of rankings.

The motivation for the critique of audiology reported by PCAST and NASEM was to evaluate cost and access to care as barriers to hearing aid use (Donahue et al., 2010). The OTC regulations prioritized these values, although not as highly as the critique documents. In the OTC regulatory documents, access to care is ranked 4/18 and cost is ranked 7/18 compared to 1/18 and 3/18, respectively, in the critique documents. This is evidence that OTC regulation generally addressed the values that motivated its creation.

The top 3 most frequently coded values in the critique documents were those that supported the creation of OTC hearing aids: access to care, autonomy, and cost. In the critique documents, the introduction of OTC hearing aids was intended to improve access and affordability and to support autonomy by making it possible for people to self-administer hearing health care. This contrasts with the three values most coded in the OTC regulations: ease of use, accuracy, and safety. Ease of use arguably supports autonomy consistent with the values of the critique documents, reflecting the need for devices to be used independently without a hearing health care professional. However, accuracy and safety reflect concerns that are not aligned with the critique documents' focus on reducing barriers to hearing aid use. In the OTC regulations, the high ranking of accuracy was due to the amount of text dedicated to electroacoustic requirements and technical specifications that must be met by a device prior to its registration as an OTC hearing aid. Examples from the final FDA regulations include limits on maximum output (Output Sound Pressure Level 90, OSPL90), the full-on gain value, output distortion control, and self-generated noise levels. The high ranking of safety likely reflects the FDA's core responsibility of establishing the safety of a medical device. According to the FDA, “there is reasonable assurance that a device is safe when it can be determined, based upon valid scientific evidence, that the probable benefits to health from use of the device for its intended uses and conditions of use, when accompanied by adequate directions and warnings against unsafe use, outweigh any probable risks” (21 CFR 860.7). Ensuring safety and accuracy in the implementation of OTC hearing aids is an obvious benefit to consumers but will add to the cost of the devices. Safety and accuracy are values that do not directly support the goal of reducing barriers to hearing aid use and prioritizing safety and accuracy above access and affordability reflects differential value prioritization between critique and regulatory documents.

The prioritization of equity and evidence-based practice was elevated in critique documents relative to regulatory documents. In the context of hearing health care, equity is defined as “fairness in treatment or outcomes” (Menon et al., 2023). Thus, the high prioritization of equity in the critique documents reflects the goal of ensuring that individuals receive effective hearing health care regardless of their background or circumstances. Equity was ranked 5/18 in critique documents, contrasted with 10/18 in the regulatory documents. The low priority of equity in OTC regulatory documents, on the other hand, represents a potentially important values conflict between the intentions of the authors of the critique and regulatory documents. Devices and services consistent with the values of the regulatory documents could reinforce existing inequities in the hearing health care system.

Evidence-based practice was ranked 4/18 in critique documents, which promoted expanding hearing health care research and the translation of this research to patient care. In contrast, OTC regulations ranked evidence-based practice 12/18. Large sections of the NASEM report (e.g., Chapter 3: Health Care Services: Improving Access and Quality) were devoted to exploring ways in which evidence-based practice can improve quality in the provision of care by hearing health care professionals, stating “evidence-based clinical practice guidelines and standards of practice can be used to educate health professionals, inform practice patterns, and facilitate widespread adherence to best practices.” The PCAST report listed the lack of adherence to evidence-based practice by hearing health care professionals as a reason for eliminating the need to obtain hearing aids through a provider, stating “studies of dispensers have found that average dispensing rates of various hearing-aid features do not follow evidence-based practice (EBP) guidelines.” Both documents criticized the poor adherence to evidence-based practice by hearing health care professionals and used this argument to support the introduction of an OTC model. Deprioritizing evidence-based practice risks suboptimal hearing health care outcomes but may support the broader goal of getting a large quantity of affordable products on the market. Disagreement between critique and regulatory documents reflects difficulty balancing the cost and importance of evidence-based practice.

### A Challenge to the Values of Traditional Audiology

The second objective of this study was to determine if the introduction of OTC hearing aids represents a challenge to the values of traditional audiology. Overall, there was agreement between the values found in documents representing traditional audiology and the values found in documents that represent OTC hearing aids. Seventeen of the 18 values identified in traditional audiology documents were also identified in OTC documents, suggesting a fundamental similarity in values between the two models. After the initial analysis revealed that the rank order of values in OTC critique and regulatory documents differed, rankings for each set of documents were compared separately to traditional audiology documents. Calculation of Kendall's τ revealed no statistically significant correlation between the rank order of values in traditional audiology documents to either critique or regulatory documents, consistent with a difference in rank order but not a reversal (negative correlation). Kendall's rank distance test showed that 50% of all code pairings were misaligned between rank order lists of values found in traditional audiology and OTC regulatory documents, consistent with a random reshuffling of the lists, and 65% of code pairings were misaligned between traditional audiology and critique documents, indicating greater disagreement than would be expected from random chance. Although they shared most of the same values, results were consistent with a reprioritization of values rankings for traditional audiology compared to both sets of OTC documents.

The goal of creating the OTC hearing aids category was to improve accessibility and affordability of amplification devices. The values of cost and access to care were central to critique documents, which recommended the introduction of OTC hearing aids to address barriers for individuals who may have previously been hesitant or unable to seek care. In contrast, traditional audiology ranked access (15/18) and cost (17/18) among the least important values. By shifting access and cost from near the bottom of the list in traditional audiology to the top of the list, the critique documents represent a direct challenge to the values of traditional audiology.

The most striking difference between the values of traditional audiology and OTC was in values related to patient benefit. Subjective benefit, the benefit from treatment that is perceived by the patient, ranked 1/18 in traditional audiology documents. Objective benefit, the benefit from treatment measured as improved performance on a task, was ranked toward the top at 6/18 in traditional audiology documents. The critique documents clearly did not prioritize these values, ranking subjective benefit 11/18 and objective benefit 16/18. In OTC regulatory documents, subjective benefit was in the bottom three values (14/18) and objective benefit was the only value with zero coding references (18/18). The stated intent of the FDA in creating the OTC regulations to ensure safety and efficacy (U.S. FDA, 2021). When the efficacy of an intervention is evaluated in traditional audiology, the metrics used typically measure subjective or objective benefit, or both, as reflected in the high ranking of these values in audiology clinical practice guidelines and questionnaires (Menon et al., 2023). Subjective metrics typically assess changes in perceived activity limitation, participation restriction, or quality of life, and objective metrics typically assess speech perception in noise. One major implication of the shift away from patient benefit is that the efficacy of OTC hearing aids will not necessarily be evaluated based on the same criteria used to evaluate audiology interventions. OTC hearing aids may instead be evaluated by the values prioritized by either the critique documents or both critique and regulatory documents, such as cost, access to care, and ease of use. One can argue that barriers to access and affordability have been effectively reduced if there are readily available devices that people can afford, even if those devices provide little subjective or objective benefit. The shift away from prioritizing subjective and objective benefit raises concerns about the impact of OTC hearing aids on the overall quality of hearing health care.

OTC hearing aids are only relevant to adults with perceived mild-to-moderate hearing difficulty. Adults between the age of 50 and 69 years who have mild hearing difficulty represent the largest population of American adults with unmet hearing health care needs (Humes, 2023). Thus, an OTC model targeting this specific patient population could provide an alternative hearing health care choice for millions of Americans with mild-to-moderate hearing difficulty. However, individuals with severe hearing loss, children, and those who cannot reasonably expect to self-fit hearing aids will not benefit from any improvement in access and affordability from the OTC model. Although the reprioritization of values in the OTC model may meet the needs of some individuals, addressing issues of access and affordability for all individuals will, according to the audiology critique documents, require reprioritizing these values within traditional audiology. Access and affordability (cost) are values in traditional audiology and, although they are low in the values prioritization, there are ongoing efforts targeting these values. Access and affordability are being addressed by other changes in audiology that are occurring parallel to the introduction of OTC hearing aids, such as the expanded use and availability of telehealth solutions (Bush & Sprang, 2019; Muñoz et al., 2021) and proposed changes to Medicare coverage (Lin et al., 2022). The success of these changes may depend on the priorities of the methods used to evaluate them, whether metrics of access and affordability are used rather than metrics of individual benefit that are traditionally used in audiology. Outside of the OTC model and the large, albeit limited population it targets, the issues with hearing health care service delivery identified by the critique documents can be addressed by targeting the values of access and affordability.

### Limitations

A limitation of the current study is the operational definition of values prioritization from the frequency of coded text references. We operationalized the rank order priority of values by counting the number of coded references to each value in the texts and verified that other methods of ordering coding references did not change the rank order. The limitation of this approach is that it is possible that some values are a high priority and yet not mentioned frequently in the documents. The act of ranking values in a definitive order is inherently challenging. Different methodologies, such as conducting surveys among audiologists or other stakeholders, might produce distinct rank orders based on their perspectives and priorities.

The purpose and motivation of the selected documents can significantly influence the resulting rank order. For example, FDA policy dictates many issues that must be addressed in establishing a medical device, so the authors of that document were not free to elaborate on issues they considered most important, unlike the authors of the critique documents. Nevertheless, FDA policy reflects a values system that directly influenced the regulatory definition of OTC hearing aids. The values prioritization in the FDA document reported in this study was consistent with the fact that the document was the product of a regulatory process with multiple constraints rather than the opinion of its authors. In reference to authors of the critique documents, the PCAST group was largely independent of the field of hearing health care, while the NASEM groups incorporated diverse stakeholders representing audiology, academia, industry, individuals who are deaf or hard of hearing, and others. Their document content, topic selection, and language reflect their values regarding hearing health care. Nevertheless, the validity of this work depends on the selection of specific documents and the rank order of values in these documents as representative of the prevailing values of the associated model.

### Future Directions

OTC hearing aids represent an opportunity to expand the market for hearing aids for adults with perceived mild-to-moderate hearing loss. Previous research shows that consumers tend to prefer engaging with services and products that align with their values (Vinson et al., 1977). The results of this study indicate that the OTC hearing aids model challenges the values of the traditional audiology model, which may provide an alternative mode of treatment that respects and aligns with a wider range of personal values and preferences. For example, OTC hearing aids may be a desirable hearing health care solution for consumers who highly value ease of use, safety, and accuracy. The critique and regulatory documents analyzed here were foundational to the OTC model but do not represent the present and future implementation of OTC hearing aids. Moving forward, traditional audiology and OTC hearing aids may work synergistically to reduce barriers to hearing health care. Audiologists may incorporate OTC hearing aids into their clinical practice by offering them as a cost-effective and accessible point of entry for patients with mild-to-moderate hearing impairment. As OTC and traditional audiology continue to advance in tandem and intersect, new values prioritizations may emerge to better serve a diverse range of individuals with different hearing abilities. Future work should focus on patient-centered outcomes by eliciting values from all stakeholders including those who could benefit from amplification but have not yet decided to seek care. Mismatches between the values of patients and the systemic values of hearing health care could explain the apparent nonuse of resources among those who could benefit. Leveraging VSD methodology will allow us to better understand the values of those who experience hearing difficulty and to develop hearing health care solutions that reflect the values of specific populations, including groups that are underserved by traditional audiology. OTC hearing aid regulations facilitate the use of VSD to bring products and services to market that are consistent with the values of underserved patients.

## Conclusions

This study tested the hypothesis that the introduction of the OTC hearing aid model represents a challenge to the values of traditional audiology. We found that the values of documents representing the introduction of the OTC model highly prioritized values consistent with reducing barriers to access and affordability of self-administered hearing health care, in contrast to the low priority of these values in traditional audiology. Elevating these values was consistent with the goal of reducing barriers to the use of hearing aids. Values highly prioritized by traditional audiology—subjective and objective benefit—were downgraded to a low priority in OTC documents. Although the reprioritization of values may benefit some people who are underserved by the current model, it is important to consider the critique of traditional audiology more broadly and explore other solutions to address the remaining barriers to access and affordability. Further research is needed to develop new solutions that align with the values of underserved patients, parallel to or facilitated by the OTC model.

## Data Availability Statement

The data sets generated and/or analyzed during this study are available from the corresponding author on reasonable request.
